# Wall-Normal Variation of Spanwise Streak Spacing in Turbulent Boundary Layer With Low-to-Moderate Reynolds Number

**DOI:** 10.3390/e21010024

**Published:** 2018-12-31

**Authors:** Wenkang Wang, Chong Pan, Jinjun Wang

**Affiliations:** Institute of Fluid Mechanics, Beihang University, Beijing 100191, China

**Keywords:** turbulent boundary layer, low speed streaks

## Abstract

Low-speed streaks in wall-bounded turbulence are the dominant structures in the near-wall turbulent self-sustaining cycle. Existing studies have well characterized their spanwise spacing in the buffer layer and below. Recent studies suggested the existence of these small-scale structures in the higher layer where large-scale structures usually receive more attention. The present study is thus devoted to extending the understanding of the streak spacing to the log layer. An analysis is taken on two-dimensional (2D) wall-parallel velocity fields in a smooth-wall turbulent boundary layer with $Re_{\tau }$ = 440∼2400, obtained via either 2D Particle Image Velocimetry (PIV) measurement taken here or public Direct Numerical Simulation (DNS). Morphological-based streak identification analysis yields a Re-independent log-normal distribution of the streak spacing till the upper bound of the log layer, based on which an empirical model is proposed to account for its wall-normal growth. The small-scale part of the spanwise spectra of the streamwise fluctuating velocity below $y^{+}$ = 100 is reasonably restored by a synthetic simulation that distributes elementary streak units based on the proposed empirical streak spacing model, which highlights the physical significance of streaks in shaping the small-scale part of the velocity spectra beyond the buffer layer.

## 1. Introduction

Low-speed streaks in wall-bounded turbulence, which were first observed by Hama and Nutant [1], Ferrell et al. [2], refer to narrow strips of low-momentum coherent motions extending lengthwise in the streamwise direction. These structures populate in the near-wall region, present quasi-regular distribution along the spanwise direction, and are always accompanied by trains of quasi-streamwise vortices with shorter length located in higher layer [3,4]. The origin of these streaks was attributed to the lift-up of low-momentum fluids from the wall under the induction of streamwise vortices [3,5,6,7,8,9], which transfers the energy from mean shear to turbulent fluctuations [10,11,12] and can be mathematically explained by a transient growth of three-dimensional (3D) disturbances due to the non-orthogonal eigenmodes in the linearized Navier-Stokes operator [13,14,15,16].

Low-speed streaks have been widely accepted as the building block of the inner-layer turbulent self-sustaining cycle [4,17,18,19,20]. The so-called bursting process, which usually denotes the whole dynamic process of the streak lift-up, oscillation and breakdown [19,21,22,23], was found to contribute to all the turbulent production and a large portion of the Reynolds stress generation in the buffer layer and below [17,19,22,24]. The generation and self-sustaining of near-wall streamwise vortices can be well explained by a streak transient growth mechanism [4], which was supported by observations that streak breakdown leads to the generation of either streamwise vortices or hairpin vortices dependent on the symmetry of the streak perturbation [25,26,27]. Hwang and Bengana [23] and Hwang et al. [28] recently reported a self-sustaining process of attached eddies in the log layer and above, in which streaks and streamwise vortices with various length scales evolve in a way similar to those in the near-wall region.

One of the ‘old’ issues related to low-speed streaks is their spanwise length scales. Note that in early wall-parallel flow visualizations [29,30], streamwise vortices were not differentiated from streaks. These two tightly-related structures contribute equivalently to the spanwise spectra of *u* component fluctuating velocity, as has been evidenced in Hwang [8] and Hwang and Bengana [23]. The spanwise spacing of neighboring streaks λ (abbreviated as streak spacing in the following) thus serves as a typical measure of the lateral length scale of near-wall structures. It is well known that in the buffer layer and below, the mean streak spacing scaled by inner variables is ${\bar{\lambda }}^{+}=\bar{\lambda }u_{\tau }/\nu ∼O\left(10^{2}\right)$ ($u_{\tau }$ is the friction velocity and ν the kinematic viscosity), and grows with respect to the wall-normal height $y^{+}$ ($y^{+}=yu_{\tau }/\nu$) [29,31,32,33,34,35]. An asymptotic linear scaling ${\bar{\lambda }}^{+}∼2y^{+}$ beyond $y^{+}$=10 was reported by Nakagawa and Nezu [36], who attributed it to the streak pairing process. Smith and Metzler [30] further suggested that merging and intermittency of streaks are responsible for the increase of ${\bar{\lambda }}^{+}$ in the region of $10<y^{+}<30$.

Previous studies examining the streak spacing using different methods are summarized in Table 1. As can be seen, most of them focused on the streak spacing in the near-wall region and suggested a Re-independency of the wall-normal growth of ${\bar{\lambda }}^{+}$ below $y^{+}$ = 30. This idea is consistent with the traditional viewpoint that near-wall energetic dynamics are independent of outer region, which is supported by both a minimum turbulent channel DNS [19,37] and a turbulent channel DNS with large-scale motions being artificially removed [8,28,38]. However, the existence of large- and very large-scale motions (LSMs and VLSMs) in outer region, which significantly affect the production of Reynolds shear stress (RSS), turbulent kinetic energy (TKE) and skin friction [39,40,41,42,43,44,45,46,47,48,49], forms a high-Re effect [50,51] by the so-called outer-layer influence. Rao et al. [21] was one of the first to experimentally show that the bursting frequency of near-wall cycle scales on the boundary layer thickness δ, which implies that large scales exert influence in the near-wall region. Bradshaw and Langer [52] reported a Re-dependency of the strength of near-wall streaks, which was recently deemed as an amplitude modulation of small-scale fluctuations by LSMs or VLSMs [50,53,54,55,56,57,58,59]. To our regards, the amplitude modulation does not conflict to the invariance of the length scale of small-scale coherent motions. Nevertheless, spectral analysis by Hoyas and Jiménez [60], Jiménez and Hoyas [61] and Hwang [8] all suggest a Re-dependency of the energetic small scales in spectral domain.

The value of studying the wall-normal variation of the lateral scale of small-scale streaks lies in the following considerations. First, the attached-eddy hypothesis [62,63,64,65,66] implies a linear growth of the spanwise length scale of energy-containing motions. Various scalings, i.e., $y\approx 1\lambda_{z}$ in Tomkins and Adrian [41], $y\approx 1/3\lambda_{z}$ in Del Álamo and Jiménez [11] and $y=0.1\lambda_{z}$ in Hwang [67], have been proposed to characterize the wall-normal growth of the lateral length scale $\lambda_{z}$ of certain kind of large-scale structures. In addition, Baars et al. [68] recently identified a self-similar wall-attached structure whose streamwise/wall-normal aspect ratio is $\lambda_{x}/y\approx 14$. None of these scalings seems to be suitable for small-scale ones. Indeed, whether or not these small-scale structures present an attached-eddy behavior is still unclear. Study on this issue might promote the understanding of how small energetic scales originated from the near-wall region behave in higher flow layers and what kind of influence large-scale structures exert on them. Secondly, to inhibit streak-centered near-wall dynamics via riblet [69], discrete roughness elements [70] or active wall actuator [71], the streak spacing is a key parameter to be known in advance. Moreover, large-eddy simulation (LES) might get improved if the spanwise distribution of streaks in the near-wall region can be modeled correctly.

Based on these reasons, the present work is devoted to studying the streak spacing from the buffer layer to the upper bound of the log layer at low-to-moderate Re. One may argue that streaks only populate in the buffer layer and below, as has been stated by Smith and Metzler [30]: Due to the streak merging and coalescence event, ‘for $y^{+}\geq 30$ streak identification becomes very uncertain, such that a process of systematic visual streak counting becomes too subjective’. To our knowledge, this statement only stresses the difficulty in detecting streaks in higher layers. Ganapathisubramani et al. [42] identified eddy packets from PIV measured wall-parallel velocity fields via feature extraction algorithm, the most probable length and width of these structures were found to follow inner scaling even in the log layer, with magnitudes comparable to those of near-wall streaks (see Figure 3 in Ganapathisubramani et al. [42] for illustration).

Hwang [8] took a numerical experiment to show that near-wall streaks and streamwise vortices can survive in outer layer if large-scale motions in the log layer and the wake region are removed. The velocity-vorticity correlation structure in turbulent channel flow at $Re_{\tau }\approx 180$, as recently identified by Chen et al. [77], well captures the geometrical feature of near-wall streaks and streamwise vortices, its spanwise width follows a scaling of $\lambda_{z}{}^{+}=0.31y^{+}+30.3$ till $y^{+}\approx 140$. Moreover, Lee et al. [48] attributed the primary source of the generation of outer-layer LSMs as the growing and merging of low-speed streaks which seem to lift from the near-wall region, which was supported by a DNS study of a minimum turbulent channel flow at low Re [78]. These studies imply the existence of streaks in higher layers. Here, we refer the term ‘streak’ as a generalized branch of small-scale structures, which share geometric and kinematic similarity with near-wall streaks and streamwise vortices. Note that LSMs and VLSMs are still streak-like, but are not included in this terminology due to their rather large length scale.

To study the streak spacing in a turbulent boundary layer, 2D velocity fields in multiple wall-parallel planes either measured by 2D planar PIV or sliced from 3D DNS dataset are analyzed. The studied Reynolds number covers a range of $Re_{\tau }$ = 440∼2430. Section 2 gives a brief description of the PIV/DNS dataset. Section 3 provides statistical evidence for the existence of small-scale streaks in flow layer beyond the buffer region. Section 4 deals with a morphological streak identification analysis, a log-normal distribution of the streak spacing with less Re-dependency is observed, and an empirical model is developed to account for its wall-normal growth from the buffer layer to the upper bound of the log layer. Finally, a simplified synthetic test is taken in Section 5. It is found that by only considering the distribution of spanwise-spaced streaks, the small-scale part of the spanwise spectra of the streamwise fluctuating velocity can be fairly well restored till the lower bound of the log layer. Concluding remarks are then given in Section 6.

## 2. Description of the PIV/DNS Dataset

### 2.1. Experiment Facilities and PIV Measurement Details

In the present study, both PIV-measured 2D wall-parallel velocity fields and DNS-obtained 3D volumetric velocity fields of a smooth-wall turbulent boundary layer are analyzed. The PIV dataset includes two configurations. One has a small field-of-view (FOV) comparable to most of the previous studies, and the other achieves a rather large FOV (on the order of δ) to clarify the effect of limited FOV on the streak spacing statistics. In the following, x/y/z denotes the streamwise/wall-normal/spanwise direction, and u/v/w the corresponding fluctuating velocity component.

For the first measurement, a flat-plate turbulent boundary layer was developed on the bottom wall of the test section of a low-speed recirculating water channel in Beihang University. The test section of this facility is made of hydraulic-smooth glass and has a size of 3 m in length, 0.7 m in height, and 0.6 m in width. With a typical free-stream velocity $U_{\infty }$ = 0.2 m/s, the free-stream turbulence intensity is about $T_{u}=0.5\%$. Boundary layer transition was triggered by a tripping wire with a diameter of 3 mm placed at 0.1 m downstream the test section inlet. The sampling station located at 2.2 m downstream the tripping wire, where the boundary layer develops to full turbulence with satisfying zero-pressure-gradient (ZPG) condition. By changing $U_{\infty }$, three frictional Reynolds number $Re_{\tau }=u_{\tau }\delta /\nu$ = 444, 761 and 1014 were achieved. They are labeled as SE1∼SE3 in Table 2, with ‘S’ short for small FOV and ‘E’ for experiment.

The large FOV measurement was performed in a large low-speed recirculating water tunnel in Beihang University. This facility has a main test section with a size of 18 m in length, 1.2 m in height, and 1 m in width, and the typical $T_{u}$ is about 0.8% when $U_{\infty }$ = 0.5 m/s. A flat plate with a length of 15 m was vertically positioned in the main test section to develop a thick turbulent boundary layer. This flat plate was assembled from 5 hydraulic-smooth Acrylic plates with lengths of 3 m, widths of 1 m, and thicknesses of 20 mm. Its leading edge had a 4:1 half-elliptical shape to avoid local flow separation. The working surface has a distance of 0.75 m from the tunnel’s side wall. For a typical boundary layer thickness δ< 0.2 m or about 25% of the gap, the effect of the side wall is negligible. The water depth was 1.0 m, the wall-parallel PIV sampling region had a vertical span of about 0.268 m and was centered at 0.47 m below the free water surface and 0.53 m above the bottom wall, far enough to neglect the free-surface/bottom-wall effect. A tripping wire with a diameter of 3 mm was glued onto the working surface at 0.4 m downstream the leading edge. The PIV sampling station was 12 m downstream. More details of the setup of this measurement can be found in Wang et al. [79]. Two cases with $Re_{\tau }$ = 1135 and 2431 were measured, denoted as LE1 and LE2 in Table 2 (‘L’ for large FOV). Note that due to the long distance of the development, the boundary layer in the measurement station suffered a minor favorable pressure gradient (FPG), the acceleration parameter *K* ($K=\left(\nu /U_{\infty }^{2}\right)dU_{\infty }/dx$) was $0.4\times 10^{-7}$∼$0.5\times 10^{-7}$. According to Harun et al. [80], a slight FPG condition will not significantly affect the energetic dynamics of large-scale structures in the outer region but only slightly increase their amplitude modulation degree to near-wall small-scale ones. We thus infer that the present minor FPG condition will not significantly bias the streak spacing statistics from other ZPG cases, this inference will be evidenced later.

**Figure 1:**
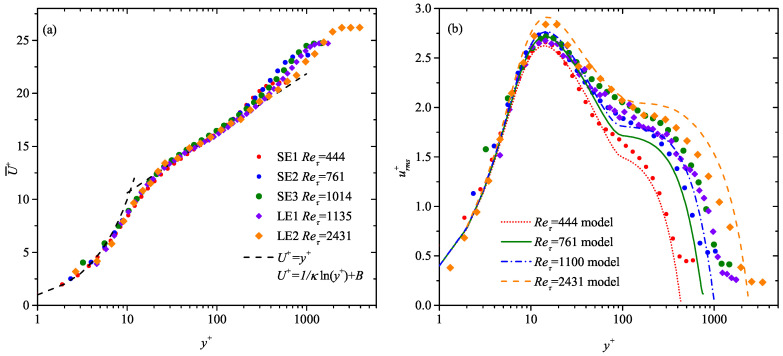
Wall-normal variation of (**a**) the mean streamwise velocity ${\bar{U}}^{+}\left(y^{+}\right)$ and (**b**) the streamwise velocity fluctuation intensity $u_{rms}^{+}\left(y^{+}\right)$ in the SE and LE cases. Straight dashed lines in (**a**) indicate the linear law and the log law, respectively. Curves in (**b**) are $u_{rms}^{+}\left(y^{+}\right)$ predicted by the empirical model of Marusic and Kunkel [83]. The present cases are represented by solid markers listed in Table 2. The same legend will be used in the following unless mentioned specifically.

Figure 1 shows the wall-normal profiles of both the mean streamwise velocity ${\bar{U}}^{+}\left(y^{+}\right)$ and the streamwise velocity fluctuation intensity $u_{rms}^{+}\left(y^{+}\right)$ obtained by a side-view 2D PIV measurement in *x*-*y* plane for all the SE and LE cases. Note that the frictional velocity $u_{\tau }$ are estimated by the Clauser fit of the ${\bar{U}}^{+}\left(y^{+}\right)$ profiles with κ = 0.41 and *B* = 5.0 [81,82]. The empirical model of $u_{rms}^{+}\left(y^{+}\right)$ in Marusic and Kunkel [83] is supplemented in Figure 1b for a comparison. Figure 1b evidences that the minor FPG condition in the LE cases only slightly suppresses $u_{rms}$ in the outer region. Table 2 summarizes the characteristic boundary layer parameters, most of which in the SE and LE cases, i.e., the shape factor *H* and the inner-scaled edge velocity $U_{\infty }^{+}$, are consistent with those in the canonical ZPG turbulent boundary layers well studied in the past [84,85,86,87,88].

Two-dimensional PIV was used to obtain instantaneous 2D velocity fields in multiple wall-parallel *x*-*z* planes. The flow field was seeded with hollow glass beads whose median diameter was 10 μm and density 1.05 g/mm${}^{3}$, and was illuminated by a double-pulsed laser sheet with thickness of about 1 mm issued from a Nd:YAG laser generator (Beamtech Vlite-500, Beijing, China) at energy output of 200 mJ/pulse. For the small-FOV LE cases, one CCD camera (Imperx ICL-B2520M, Boca Raton, FL, USA) with a resolution of 2456×2048 pixels was used for image recording. To guarantee a comparable inner-scaled magnification, a Nikkor 50 mm f/1.8D lens was used for the SE1 case and a Tamron 90 mm f/2.8D lens for the SE2 and SE3 cases. The FOV was 85×101 mm${}^{2}$ (streamwise span ΔX× spanwise span ΔZ) and 36×43 mm${}^{2}$, respectively. The corresponding magnification were 0.24, 0.2 and 0.285 wall units/pixel. In the large-FOV LE cases, 8 synchronized CCD cameras (Imperx ICL-B2520M) mounted with Nikkor 50 mm f/1.8D lens were arranged in a 4×2 array with 10∼15 mm overlap in the image plane, and jointly provided a total FOV of 636 ×268 mm${}^{2}$. The magnification was 0.39 and 0.96 wall units/pixel in the LE1 and LE2 case, respectively. The inner-scaled FOV are listed in Table 2. To explore the effect of the FOV truncation effect (in Section 4.1), velocity fields in the LE2 case will be sliced to a FOV span $\Delta Z^{+}$ = 1500, the same to that of the LE1 case when necessary. In both PIV configurations, around 3600 pairs of particle images were recorded at each measurement plane. The sampling repetition rate was 7.5 Hz in the SE cases and 5 Hz in the LE cases. The whole sampling duration $Tu_{\tau }/\delta$, as listed in Table 2, was large enough for the convergence of the second-order statistics of the fluctuating velocity.

An optical flow solver based on the Lucas-Kanade algorithm was used to calculate 2D velocity fields from particle image pairs via GPU acceleration [92,93]. The interrogation window in the final pass was 48×48 pix with an overlap of 75%. The spatial resolution was about 6 wall units/vector in the SE cases and increased to 9 and 23 wall units/vector in the LE cases. The straddle time within the image pairs was selected to keep the maximum particle offset around 14∼16 pixels in the image plane. The relative error of the velocity measurement was estimated to be less than 1%.

The optical system was mounted on a linear stage, allowing the wall-normal offset of the laser sheet at a resolution of 0.01 mm. A comparison of the ${\bar{U}}^{+}\left(y^{+}\right)$ and $u_{rms}^{+}\left(y^{+}\right)$ profiles obtained by the wall-parallel PIV measurement with those by side-view measurement showed satisfying consistency (not shown here for simplicity). The uncertainty of the laser sheet positioning was estimated to be around $\sigma_{y}^{+}$ = 1∼3. In the large-FOV LE cases, a 45° inclined reflective mirror with length of 100 mm and width of 10 mm was positioned at 0.8 m downstream the end of the FOV, it reflected the laser sheet towards upstream to provide a large illumination extent without substantially affecting the upstream flow field. Cylindrical lenses with long focus length were used to keep the laser sheet thickness be around 1 mm over a distance of 2 m. The wall-parallel condition was checked by keeping the variation of the height of the laser sheet less than 0.5 mm over a distance of 1.5 m. Table 3 summarizes the wall-parallel planes being measured. According to Klewicki et al. [94] and Marusic et al. [82], the upper bound of the log layer can be estimated as around y/δ = 0.15. The planes above this height are labeled with asterisks ∗. Note that the lowest measurement position was constrained by the laser sheet thickness, the wall reflection and the width of the immersed mirror, and was $y_{min}$ = 3 mm above the wall for the SE cases and $y_{min}$ = 5 mm for the LE cases.

### 2.2. DNS Dataset

Four DNS datasets of a spatially developing turbulent boundary layer over a smooth wall are also analyzed. As shown in Table 2, the LD0 case (‘L’ for large FOV and ‘D’ for DNS) with $Re_{\tau }$ = 440 was obtained by Simens et al. [89], and the LD1∼LD3 cases with $Re_{\tau }$ = 1100∼2000 were obtained by Sillero et al. [90,91]. Readers can refer to Simens et al. [89], Sillero et al. [90,91], Borrell et al. [95] for detailed description about these DNS datasets.

Each LD case analyzed here contains 20 snapshots of instantaneous 3D volumetric velocity fields, which are available online (http://torroja.dmt.upm.es/ftp/blayers/). Planar velocity fields in multiple *x*-*z* planes (as indicated in Table 3) were sliced from these snapshots with streamwise extent of 2000 wall units and spanwise extent covering the whole simulation domain (i.e., 6000 wall units for the LD0 case and about 16,000 wall units for the LD1∼LD3 cases). They were then cut into smaller sections with a size of $\Delta X^{+}\times \Delta Z^{+}$ = 2000 × 1500, making $\Delta Z^{+}$ comparable to those in the LE cases. This formed an ensemble of about 80 realizations in the LD0 case and 200 realizations in the LD1∼LD3 cases. As will be shown in Section 4.1 and Appendix B.3, the ensemble size is large enough for the convergence of the probability density function (PDF) of the streak spacing in the log layer and below. One advantage of DNS dataset is that the inner-layer is fully-resolved, which provides an ideal supplement for the PIV experiment which is limited by the lowest measurement plane.

## 3. Existence of Small-Scale Streak in Higher Layer

**Figure 2:**
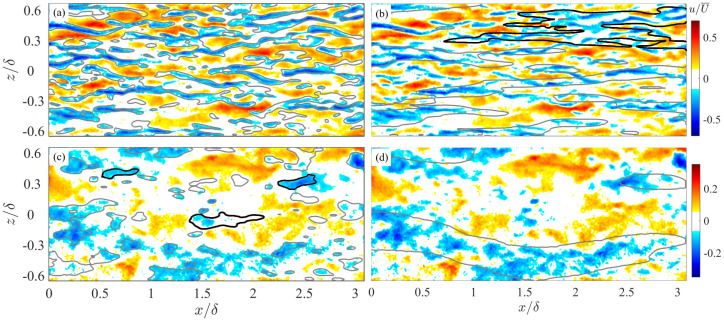
A snapshot of streamwise fluctuating velocity field $u/\bar{U}$ (pseudo-color maps) in the LE1 case at (**a**,**b**) $y^{+}$ = 28 and (**c**,**d**) $y^{+}$ = 226. Proper orthogonal decomposition (POD)-separated high- and leading-order field, i.e., $u^{H}$ and $u^{L}$, are superimposed in (**a**,**c**) and (**b**,**d**) as isolines, respectively. The solid isolines represent low-speed regions with the level of $u^{H|L}=-0.05\bar{U}$. The bold isoline in (**b**) indicates a region of the amalgamation of several small-streak streaks to form a large-scale structure in $u^{L}$, while bold isolines in (**c**) indicate streaks which are isolated from large-scale motions (LSMs) revealed in $u^{L}$.

To study the streak spacing beyond the buffer layer, the first issue to be clarified is whether they exist in higher flow layer with statistical significance. Figure 2 illustrates typical instantaneous u(x,z) fields in the LE1 case at $y^{+}$ = 28 (in the buffer layer) and $y^{+}$ = 226 (above the upper bound of the log layer). It can be visually identified that small-scale streaks and LSMs are dominant structures at $y^{+}$ = 28 and $y^{+}$ = 226, respectively. But structures with length scales far from the local most energetic scale are also observable in both flow layers.

For a quantitative description of such a multi-scale feature, a flow-field scale separation is desired. Fourier-based scale filtering was commonly used for this purpose [48,54], its limitation is the arbitrariness in the selection of the scale cutting-off threshold. Another popular method is the Empirical Mode Decomposition (EMD) and its derivatives [79,96], which empirically separates the length scales without reference to a fixed scale threshold. Nevertheless, EMD-based method usually requires a predetermined mode number, and its physical interpretation is not as clear as Fourier decomposition. In the present study, Proper Orthogonal Decomposition (POD) is used as an alternative. POD has been used as a scale-filtering tool to isolate large-scale structures from small-scale ones in wall-bounded turbulence [45,97]. In essence, it decomposes a given space-time realization V(x,t) into a linear combination of a set of orthogonal bases whose spatial and temporal dimension are fully decoupled as:

(1) $$V\left(x,t\right)=\sum_{n=1}^{N}a_{n}\left(t\right)\phi_{n}\left(x\right)=\underset{V^{L}}{\underset{︸}{\sum_{n=1}^{s}a_{n}\left(t\right)\phi_{n}\left(x\right)}}+\underset{V^{H}}{\underset{︸}{\sum_{n=s+1}^{N}a_{n}\left(t\right)\phi_{n}\left(x\right)}}.$$

In Equation (1), $a_{n}\left(t\right)$ is the time coefficient of the *n*th mode, $\phi_{n}\left(x\right)$ is the corresponding mode basis function and *N* is the total number of the POD modes. The decomposition is based on an optimal energy recovery criteria, i.e., the TKE recovery using the POD mode bases is always the best for each level of reconstruction. In this sense, POD decomposes the flow-field ensemble by energy content, in distinct contrast to the scale-based decomposition methods (FFT filtering or EMD). The multi-scale structures in wall-bounded turbulence have different TKE contribution, so that they are projected onto different POD modes.

Snapshot POD analysis [98,99] is applied to all the SE and LE cases. As a supplementary illustration, Appendix A illustrates the cumulative TKE contribution of all the POD modes, the characteristic spanwise length scale carried by each mode and the typical mode basis functions in the LE1 case. Since the POD modes are ranked by their relative TKE contribution $E_{k}=\lambda_{k}/\Sigma_{n=1}^{N}\lambda_{n}$ with $\lambda_{n}$ the eigenvalue of the *n*th mode, a cumulative energy cut-off threshold $P_{s}=\Sigma_{n=1}^{s}E_{n}$ can be set to separate all the POD modes into a leading-order group including the first *s* modes and a high-order group containing the rest N−s+1 ones. Similar to Wu and Christensen [45] and Deng et al. [97], velocity field reconstruction using these two mode groups via the right part of Equation (1) is taken. This separates the original full-order V into a leading-order $V^{L}$ and a high-order $V^{H}$. In the following, the energy cut-off threshold is set as $P_{s}$ = 0.5, i.e., $V^{L}$ and $V^{H}$ equally contribute to 50% of the total TKE. Additional tests showed that a moderate change of $P_{s}$ around 0.5 will not significantly affect the characteristic scales contained in $V^{L}$ and $V^{H}$. Appendix A further shows that all the modes with spanwise scale larger than δ are fully sorted into the leading-order group when $P_{s}$ = 0.5.

The scale-separated velocity fields are visualized as the isolines of $u^{H|L}$ = 0.05$\bar{U}$ superimposed onto the full-order *u* field in Figure 2a–d, respectively. In the near-wall region, the footprint of outer-layer LSMs can be visualized as the amalgamation/coordination of several small-scale streaks to a larger one (see the structure highlighted with bold isolines in Figure 2b for example). While in the log layer and above, $u^{H}$ captures the core regions of LSMs. Small-scale structures independent from LSMs also appear now and then in $u^{H}$ (as indicated bold isolines in Figure 2c). They have reduced streamwise extent and expanded spanwise scale if compared to the near-wall streaks.

**Figure 3:**
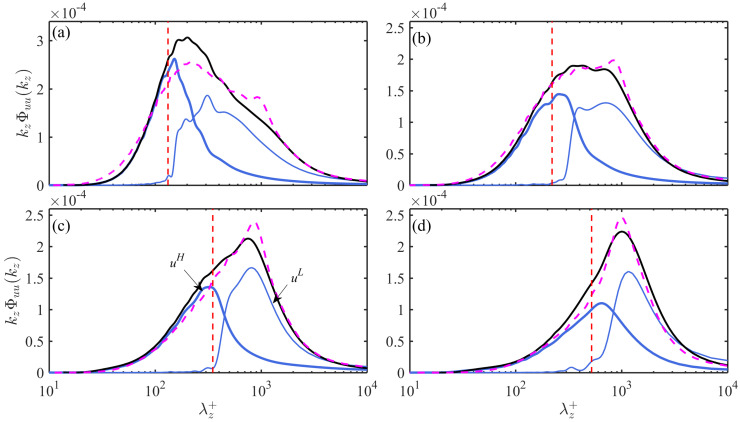
Comparison of premultiplied spanwise spectra $k_{z}\Phi_{uu}$ of the full-order *u* fields with $k_{z}\Phi_{u^{L|H}u^{L|H}}$ of the POD-separated leading- or high-order $u^{L|H}$ fields in the LE1 case at (**a**) $y^{+}\;=\;28$; (**b**) $y^{+}\;=\;57$; (**c**) $y^{+}=113$ and (**d**) $y^{+}\;=\;226$. $k_{z}\Phi_{uu}$ in the LD1 case is also given for a comparison. **―**: $k_{z}\Phi_{uu}$ of LE1; — —: $k_{z}\Phi_{uu}$ of LD1; ―: $k_{z}\Phi_{u^{L|H}u^{L|H}}$ of LE1 with $P_{s}$ = 0.5. Vertical dashed lines are the most probable streak spacing $\lambda_{mp}^{+}$ predicted by the empirical model in Section 4.2.

Figure 3 further shows the pre-multiplied spanwise spectra $k_{z}\Phi_{uu}\left(\lambda_{z}^{+}\right)$ of the LE1 case (with $Re_{\tau }$ = 1135, bold solid curves) at various $y^{+}$. Agreement with those of the LD1 case (with $Re_{\tau }$ = 1100, dashed curves in Figure 3) is observed. Both cases present a quick increase of the most energetic length scale from ${\lambda_{z}^{\Phi }}^{+}∼O\left(10^{2}\right)$ at $y^{+}$ = 28 (in Figure 3a) to $\lambda_{z}^{\Phi }∼\delta$ at $y^{+}$ = 113 (in Figure 3c). The spectra profiles $k_{z}\Phi_{u^{L}u^{L}}\left(\lambda_{z}^{+}\right)$ and $k_{z}\Phi_{u^{H}u^{H}}\left(\lambda_{z}^{+}\right)$ of the POD-separated $u^{L}$ and $u^{H}$ are also shown in Figure 3 (as thin solid curves): the inner-scaled and outer-scaled spectrum peak can be now distinguished from each other at each flow layer. This evidences that the decoupling of TKE via POD does lead to the separation of length scales. More importantly, a distinct peak always appears in the $k_{z}\Phi_{u^{H}u^{H}}$ spectra till the upper bound of the log layer. To our regards, this spectra peak is attributed to the streak-liked structures in $u^{H}$ as visualized in Figure 2, the corresponding peak $\lambda_{z}$ can thus be interpreted as the characteristic spanwise scale of these structures. This is supported by the observation that $\lambda_{z}$ of the $k_{z}\Phi_{u^{H}u^{H}}$ peak always has correspondence with the most probable streak spacing (dashed lines in Figure 3), which will be discussed in Section 4.

The prevalence of small-scale streak-liked structures in higher layers can be further evidenced by the map of two-point correlation coefficient $\bar{R}$, which was widely used in previous researches [36,41,43,53,73,91]:

(2) $${\bar{R}}_{\chi \chi }\left(r_{x},r_{z},y_{ref}\right)=\frac{\langle \chi \left(x,y_{ref},z,t\right)\cdot \chi \left(x+r_{x},y_{ref},z+r_{z},t\right)\rangle }{\sigma_{\chi }^{2}}$$

**Figure 4:**
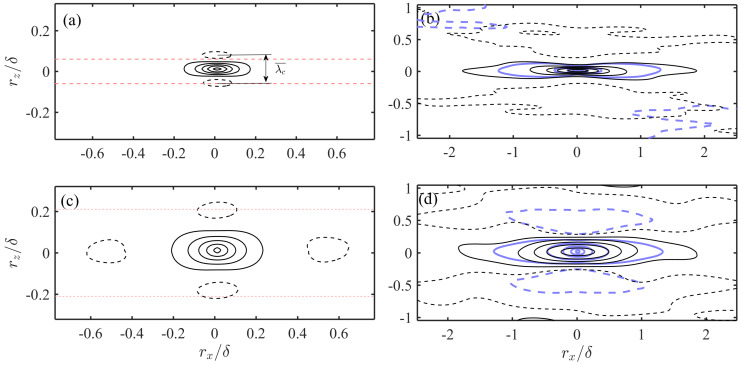
Two-point correlation map of POD-separated $u^{L|H}$ fields in the LE1 case with $P_{s}$ = 0.5. (**a**) ${\bar{R}}_{u^{H}u^{H}}$ at $y^{+}$ = 28; (**b**) ${\bar{R}}_{u^{L}u^{L}}$ at $y^{+}$ = 28; (**c**) ${\bar{R}}_{u^{H}u^{H}}$ at $y^{+}$ = 226; (**d**) ${\bar{R}}_{u^{L}u^{L}}$ at $y^{+}$ = 226. Thin solid/dashed isolines represent positive/negative correlation with contour level uniformly spaced from −0.1 to 0.9 with a gap of 0.2. ${\bar{R}}_{uu}$ of the full-order *u* fields are superimposed in (**b**,**d**) as bold solid/dashed isolines. The interval between the horizontal thin dashed lines in (**a**,**c**) indicates the most probable streak spacing $\lambda_{mp}$ predicted by the empirical model in Section 4.2.

In Equation (2), χ is *u*, $u^{L}$ or $u^{H}$, $r_{x}$ and $r_{z}$ are the x/z offset from the reference point, $\sigma_{\chi }$ is the standard deviation of χ, and 〈·〉 the average over both the spatial and temporal domain. Figure 4 plots ${\bar{R}}_{u^{H}u^{H}}$ and ${\bar{R}}_{u^{L}u^{L}}$ at $y^{+}$ = 28 and 226 in the LE1 case with $P_{s}$ = 0.5. ${\bar{R}}_{uu}$ of the full-order *u* fields are supplemented in Figure 4b,d (as bold isolines). As shown in Figure 4b,d, ${\bar{R}}_{u^{H}u^{H}}$ and ${\bar{R}}_{u^{L}u^{L}}$ in inner and outer region are both characterized as streamwise elongated structures with length scales sufficiently gaped from each other, resembling those instantaneous structures shown in Figure 2. A characteristic spanwise scale $\bar{\lambda_{c}}$ can be defined as the gap between the two negative valleys as illustrated in Figure 4a. The spanwise scale of ${\bar{R}}_{u^{L}u^{L}}$ and ${\bar{R}}_{uu}$ are both $\bar{\lambda_{c}}∼\delta$, consistent with previous studies that showed the validity of the outer scaling of ${\bar{R}}_{uu}$ even in the buffer region of high Re TBL [41,43,53]. Figure 4a,c shows that ${\bar{R}}_{u^{H}u^{H}}$ present streak-liked pattern in both $y^{+}$ = 28 and $y^{+}$ = 226. This provides a statistical evidence for the existence of small-scale streaks beyond the buffer region. Furthermore, $\bar{\lambda_{c}}$ of ${\bar{R}}_{u^{H}u^{H}}$ is much smaller than those of ${\bar{R}}_{u^{L}u^{L}}$, the the magnitude is rather close to the most probable streak spacing to be shown in Section 4.2.

## 4. Streak Spacing Based on Morphological Identification

To further study the spanwise spacing of neighboring streaks, the morphological-based streak identification algorithm proposed by [76] is used in this section with slight modification. The essence of this algorithm is to isolate low-speed streak-liked regions by binarizing u(x,z) snapshots with a pre-given velocity deficit threshold and extract their skeletons with the aid of computer vision technique. The streak spacing is then counted as the spanwise gap between two adjacent low-speed streak skeletons only if at least one high-speed streak skeleton is clapped in between. The algorithm details are given in Appendix B.1. It is stressed that this algorithm does not differentiate small-scale structures from large-scale ones, but only finds the nearest gap between two neighboring streak-liked structures. Nevertheless, Figure 2 shows that even the core region of LSMs is clustered with small-scale coherent motions, thus the streak spacing obtained by this algorithm will represent the typical spanwise scale of the smallest energetic structures.

In this algorithm, there are a set of parameters, i.e., the velocity deficit threshold, the non-streak filter, and the skeleton extracting parameters, that should be set manually. As shown in Appendix B.2, their influence on the streak skeleton extraction is rather weak, any moderate change from the selected parameter combination will only lead to a change of the statistics of the streak spacing less than 15%.

This morphological algorithm is applied to all the present studied cases. The ergodic state to account for the streak pattern variation, i.e., streak splitting or merging, is achieved by counting the streak spacing at streamwise stations gaped as $\Delta x^{+}\approx 30$ in every snapshot. This forms an ensemble with samples more than $O(10^{6})$ for the SE cases, $O(10^{7})$ for the LE cases and $O(10^{6})$ for the LD cases in the near-wall region. However, due to the reduced streak population (to be discussed in Section 4.2), the ensemble size drops to $O(10^{4})$, $O(10^{5})$ and $O(10^{4})$ in the log layer, respectively. A convergence test is taken in Appendix B.3 to show that for all the studied cases, both the PDF of the streak spacing and the related statistics get acceptable convergence till the upper bound of the log layer.

### 4.1. Streak Spacing Distribution

**Figure 5:**
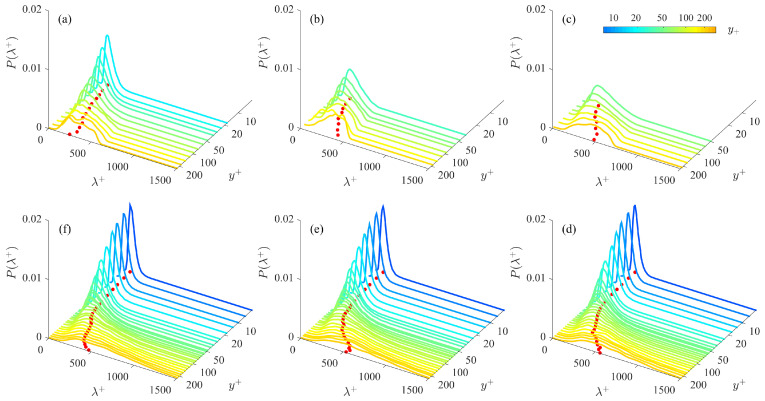
Wall-normal variation of the PDF of the streak spacing $P(\lambda^{+})$. (**a**) SE1; (**b**) SE2; (**c**) SE3; (**d**) LD1; (**e**) LD2; (**f**) LD3. The local maxima of the probability density function (PDF) are projected on the $\lambda^{+}$-$y^{+}$ plane as solid dots.

Figure 5 gives an overview of the wall-normal variation of the PDF of inner-scaled streak spacing $\lambda^{+}$ in the SE and LD cases. Every PDF profile $P(\lambda^{+})$ presents a single peak without a sign of bi-modal pattern even in the upper bound of the log layer. The long tail of $P(\lambda^{+})$ extends towards the large value side to form a left-skewed shape. The most probable streak spacing $\lambda_{mp}^{+}$ increases monotonically with $y^{+}$, while $P(\lambda_{mp}^{+})$ decreases, in together with a distinct growth of the long tail. However, a so-called ‘truncation effect’ is observed in higher layers of the SE cases: due to the limited FOV span ($\Delta Z^{+}$ = 500∼700), those events with potentially large $\lambda^{+}$ are not detected, making a remarkable shortening of the long tails of $P(\lambda^{+})$ if compared to those in the LD cases. Note that truncation effect has been inferred by Smith and Metzler [30] as ‘This result (biased streak spacing) was felt to be a consequence of the narrowness of the data window’. A detailed inspection of all the $P(\lambda^{+})$ profiles shows that for the LE and LD cases whose FOV span $\Delta Z^{+}$ = 1500 is rather large, the truncation effect is minor; while for the LE cases, once $\lambda_{mp}^{+}$ is far from $\Delta Z^{+}$, the truncation of the long tail part of $P(\lambda^{+})$ will not bias the value of $\lambda_{mp}^{+}$ but change the overall probability level.

**Figure 6:**
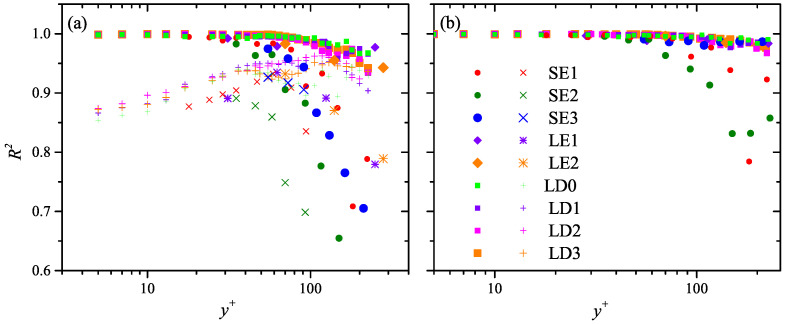
Wall-normal variation of the determination coefficient $R^{2}$ in all the studied cases. (**a**) $R^{2}$ of both the log-normal fitting via Equation (3) (solid circular, diamond, or rectangle) and the Rayleigh fitting via Equation (4) (diagonal cross, star, and cross); (**b**) $R^{2}$ of the dimensional constraint log-normal fitting via Equation (5).

As summarized in Table 1, Lee et al. [34] and Smith and Metzler [30] reported a log-normal distribution of the streak spacing $\lambda^{+}$ in the near-wall region, while an alternative Rayleigh distribution was claimed by Lin et al. [76]. These two distributions are:

(3) $$P\left(\lambda^{+}\right)=\frac{1}{\lambda^{+}\sigma \sqrt{2\pi }}\exp\left(-\frac{{\left(\ln\lambda^{+}-\mu \right)}^{2}}{2\sigma^{2}}\right),$$

(4) $$P\left(\lambda^{+}\right)=\frac{\lambda^{+}}{s^{2}}\exp\left(-\frac{\lambda^{+2}}{2s^{2}}\right),$$

with free parameters (μ, σ) and *s*, respectively. Both models are evaluated by the raw $P(\lambda^{+})$ profiles via a least-square fitting. The fitting determination coefficients $R^{2}$, as shown in Figure 6a, suggests that the log-normal model outperforms the Rayleigh model everywhere, but the former still presents a performance drop beyond $y^{+}\approx 30$, which is more prominent in the SE cases with smaller FOV span. This is attributed to the deteriorated truncation effect that begins to distort the $P(\lambda^{+})$ profiles in higher layer.

**Figure 7:**
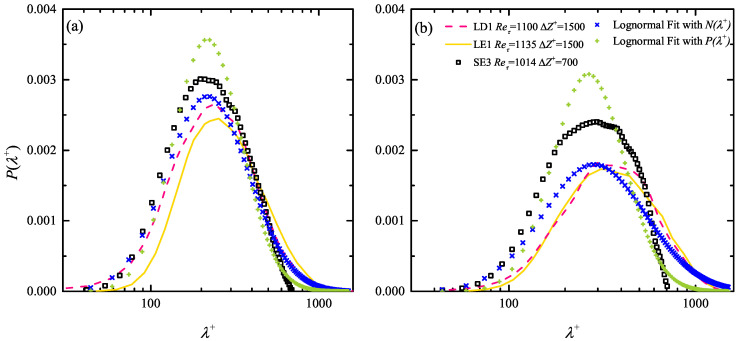
Illustration of the feasibility of the dimensional constraint log-normal fitting via Equation (5) in the SE3 case at (**a**) $y^{+}$ = 65; (**b**) $y^{+}$ = 131. Dashed and solid curves are $P(\lambda^{+})$ at similar $y^{+}$ in the LD1 and LE1 cases where the field-of-view (FOV) truncation effect are minor and $Re_{\tau }$ are similar. Rectangle markers are the raw $P(\lambda^{+})$ profiles in the SE3 case, diagonal cross markers are the estimations via Equation (5), while cross markers are the estimations via Equation (3).

A dimensional constraint log-normal fitting is proposed to compensate for the truncation effect. This fitting is based on the observation that in a non-severe truncation case where the most probable $\lambda_{mp}^{+}$ is far from the FOV span $\Delta Z^{+}$, only the long tail of $P(\lambda^{+})$ close to $\Delta Z^{+}$ is truncated, in together with the enhancement of the probability level of smaller $\lambda^{+}$ events. This is clearly seen in Figure 7 which highlights the difference in the $P(\lambda^{+})$ profiles between the SE3 case and the LE1/LD1 cases with similar $Re_{\tau }$. A log-normal fitting can then be applied to the dimensional frequency number distribution $n(\lambda^{+})$ instead of the non-dimensional probability $P(\lambda^{+})$ via

(5) $$n\left(\lambda^{+}\right)=\frac{\alpha }{\lambda^{+}\sigma \sqrt{2\pi }}\exp\left(-\frac{{\left(\ln\lambda^{+}-\mu \right)}^{2}}{2\sigma^{2}}\right),\;0<\lambda^{+}<C\Delta Z^{+}.$$

In Equation (5), only $n(\lambda^{+})$ on the left of $\lambda^{+}=C\Delta Z^{+}$ is fitted. The parameter *C* regulates the fitting range and is manually fixed as 0.8 here, i.e., the right 20% part of the $n(\lambda^{+})$ profile is rejected in this fitting. An additional free parameter α appears in Equation (5), it allows the floating of the integral area of the $n(\lambda^{+})$ profile.

The validity of this dimensional constraint fitting is illustrated in Figure 7. It shows that even in the log layer ($y^{+}\approx$ 130), the raw $P(\lambda^{+})$ profiles in the LE1/LD1 cases present Gaussian shape with satisfying symmetry in a logarithmically scaled *x* axis. In contrast, the truncation of the long tails of $P(\lambda^{+})$ of the SE3 case (hollow square markers), due to the insufficient FOV span, leads to asymmetrical profiles. The raw $P(\lambda^{+})$ profiles (hollow square markers) of the SE3 case at $y^{+}\approx$ 60 and 130 are then fitted via both canonical log-normal model (Equation (3)) and the dimensional constraint version (Equation (5)). The latter leads to a more reasonable prediction of $P(\lambda^{+})$ (diagonal cross markers) if compared to the raw profiles in the LE1/LD1 cases (dashed/solid lines) which are believed to be less affected by the truncation effect.

With this truncation compensation strategy, the performance of the log-normal model, as can be seen in Figure 6b, gets persistently improved. The enhancement of $R^{2}$ is quite remarkable for the SE cases. For the LE/LD cases, the magnitude of $R^{2}$ in higher layer ($y^{+}>$ 100) elevates above 0.98, indicating a good accordance to the log-normal model. Nevertheless, $R^{2}$ in the SE1/SE2 cases beyond $y^{+}=$ 100 is still smaller than 0.9, the reason is that the most probable part of these $P(\lambda^{+})$ profiles are rather close to ΔZ, making the full compensation of the truncation effect rather difficult.

### 4.2. An Empirical Model for Streak Spacing

Given a log-normal distribution of $P(\lambda^{+})$, the mean and the most probable streak spacing, i.e., ${\bar{\lambda }}^{+}$ and $\lambda_{mp}^{+}$, can be determined by the controlling parameters μ and $\sigma^{2}$ in Equation (3) or (5):

(6) $$\left.\begin{array}{c} {\bar{\lambda }}^{+}=e^{\mu +\sigma^{2}/2}\\ \lambda_{mp}^{+}=e^{\mu -\sigma^{2}} \end{array}.\right.$$

**Figure 8:**
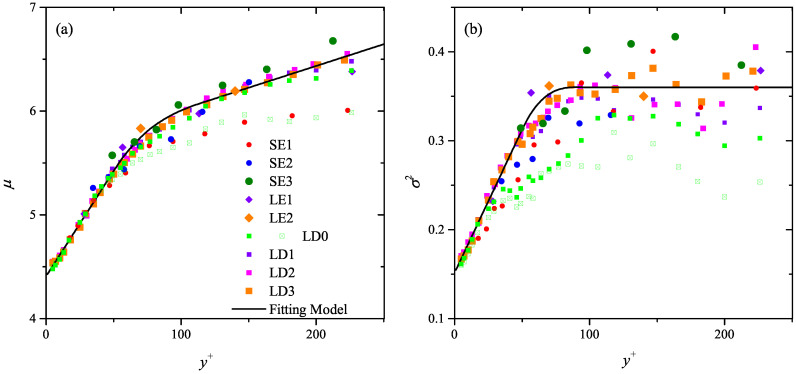
Wall-normal variation of the free parameter (**a**) μ and (**b**) $\sigma^{2}$ in log-normal distribution. Solid markers are the estimations by dimensional constraint model (Equation (5)) in all the studied cases, and bold solid lines are the two-stage linear model of Equations (7) or (8). Hollow squares with cross markers indicate the truncated LD0 case with $\Delta Z^{+}$ = 600.

Figure 8 summarizes μ and $\sigma^{2}$ in all the studied cases estimated by the dimensional constraint fitting via Equation (5). Except for the SE1 case with $y^{+}>100$, μ is independent from Re till $y^{+}\approx 220$. In contrast, $\sigma^{2}$ presents a non-negligible scattering beyond $y^{+}$=100. Note that the scattering level is $\Delta \sigma^{2}∼O\left(10^{-1}\right)$, more than one-order smaller than the magnitude of μ; therefore, its contribution to ${\bar{\lambda }}^{+}$ and $\lambda_{mp}^{+}$ in Equation (6) is comparably small. The smaller magnitude of μ in the SE1 case with $y^{+}>100$ is attributed to the inability of compensating the truncation effect when $\lambda_{mp}$ is rather close to the FOV span ΔZ. For a test, the LD0 case with a similar $Re_{\tau }$ is resampled with the same FOV as SE1, i.e., $\Delta Z^{+}$=600. The magnitudes of μ (hollow squares with cross markers in Figure 8a) are now comparable to those of the SE1 case, and $\sigma^{2}$ (in Figure 8b) also get distinctly reduced.

μ and $\sigma^{2}$ in the LD cases, which are less affected by the truncation effect, are used to construct an empirical model from the buffer layer to the upper bound of the log layer (y/δ∼0.15):

(7) $$\mu =\left\{\begin{array}{c} 0.02y^{+}+4.4,\;10<y^{+}<50\\ 4.2\times 10^{-3}y^{+}+5.60,\;100<y^{+}<\min\left(0.15\delta^{+},220\right) \end{array}\right.$$

(8) $$\sigma^{2}=\left\{\begin{array}{c} 3.2\times 10^{-3}y^{+}+0.15,\;10<y^{+}<50\\ 0.36,\;100<y^{+}<\min\left(0.15\delta^{+},220\right) \end{array}\right.$$

This empirical model includes two linear stages of $\mu (y^{+})$ and $\sigma^{2}\left(y^{+}\right)$, i.e., within $y^{+}$=10∼50 and beyond $y^{+}$ = 100, which are bridged by a cubic fitting in the middle. As shown in Figure 8, it fairly predicts $\mu (y^{+})$ and $\sigma^{2}\left(y^{+}\right)$ of the SE and LE cases till the upper bound of the log layer. Using this model, the wall-normal variation of ${\bar{\lambda }}^{+}$ and $\lambda_{mp}^{+}$ can be predicted via Equation (6).

**Figure 9:**
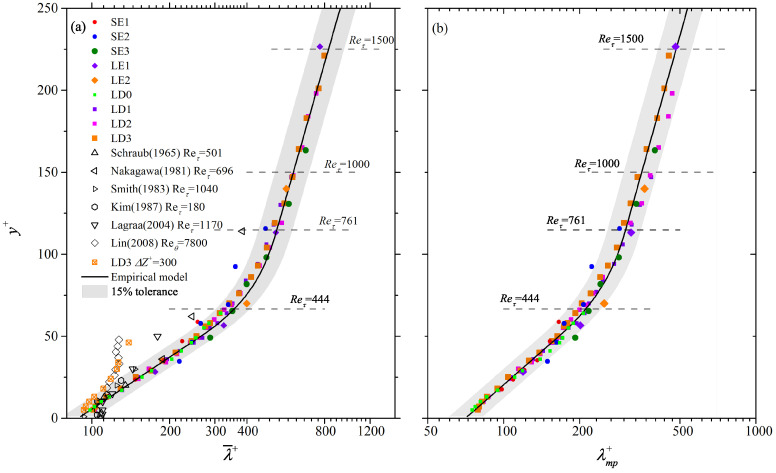
Wall-normal variation of (**a**) ${\bar{\lambda }}^{+}$ and (**b**) $\lambda_{mp}^{+}$ till the upper bound of log layer. Solid markers are the estimations by Equation (5) in all the studied cases. Only the data below the upper bound of the log layer, i.e., y/δ∼0.15 indicated as dashed horizontal lines for typical $Re_{\tau }$ in (**a**), is shown. The same in Figure 10 and Figure 11. Hollow markers in (**a**) are ${\bar{\lambda }}^{+}$ obtained by previous studies listed in Table 1. Hollow squares with cross markers indicate the truncated LD3 case with $\Delta Z^{+}$ = 300. Bold solid curves are predictions of the empirical model of Equations (6)∼(8), with shaded regions indicating a ±15% tolerance.

The validity of this empirical model can be evidenced by the following two aspects. Firstly, as shown in Figure 9, ${\bar{\lambda }}^{+}\left(y^{+}\right)$ and $\lambda_{mp}^{+}\left(y^{+}\right)$ of the SE and LE cases (solid dots), which are not used to construct this model, generally collapse onto the model’s prediction (bold solid lines) till the upper bound of the log layer. Moreover, ${\bar{\lambda }}^{+}$ in the near-wall region reported by most of previous studies [30,72,74,75] (hollow markers in Figure 9a) are also compatible with it. Two exceptions are Nakagawa and Nezu [36] and Lin et al. [76], who reported remarkably smaller ${\bar{\lambda }}^{+}$ beyond $y^{+}$ = 30. To our regards, this might be related with either the condition invoked in the conditional correlation calculation in Nakagawa and Nezu [36] or the insufficient FOV span ($\Delta Z^{+}$ = 320) in Lin et al. [76]. For the latter, the LD3 case with similar Re is resampled by $\Delta Z^{+}$ = 300, this leads to a reduced ${\bar{\lambda }}^{+}$ (hollow squares with cross markers in Figure 9a) consistent with those in Lin et al. [76].

**Figure 10:**
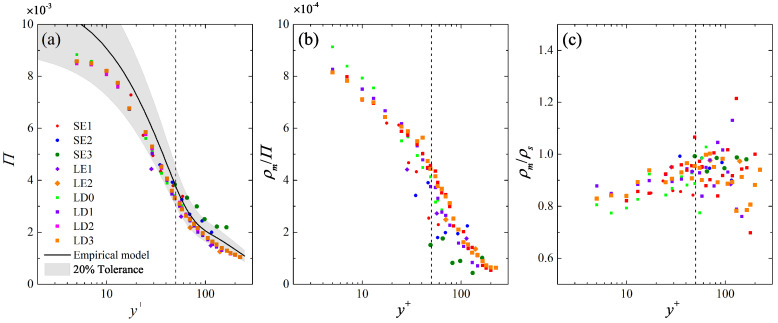
Wall-normal variation of (**a**) the mean streak population density Π; (**b**) the streak merging frequency $\rho_{m}/\Pi$ and (**c**) the ratio between the streak merging and splitting frequency $\rho_{m}/\rho_{s}$ till the upper bound of the log layer (y/δ∼0.15). In (**a**), bold solid lines are the prediction of the empirical model of Equations (6)∼(8), with the shaded regions indicating a ±20% tolerance. Vertical dashed lines indicates the flow layer of $y^{+}=50$, the same in Figure 11.

Secondly, the linear scaling of μ and $\sigma^{2}$ in Equations (7) and (8) leads to a two-stage exponential growth of ${\bar{\lambda }}^{+}$. Since the mean streak population density Π, which measures the average number of streaks per unit span, is the inverse of ${\bar{\lambda }}^{+}$, a two-stage exponential decay of Π is expected. As shown in Figure 10a, $\Pi (y^{+})$ obtained by counting the number of the identified streaks in the whole snapshot ensemble, present less scattering among all the studied cases. The general trend follows a two-sectional decay gaped at $y^{+}\approx 50$, and fairly agrees with the prediction of the empirical model till the upper bound of the log layer, again evidencing the validity of the latter.

### 4.3. Discussion on the Empirical Model for Streak Spacing

The proposed empirical model (Equations (6)–(8)) describes a Re-independent two-stage exponential growth of the mean streak spacing along the wall-normal direction, the second stage of which has a reduced growth rate beyond $y^{+}$ = 50. For a kinematic explanation of such a growth trend, the wall-normal variation of the streak amplitude $A_{s}$ is first investigated. Here, $A_{s}$ measures the peak momentum deficit within one streak. In the present study, it is simplified as the normalized local streamwise fluctuation velocity $u/\bar{U}$ on the identified streak skeleton.

**Figure 11:**
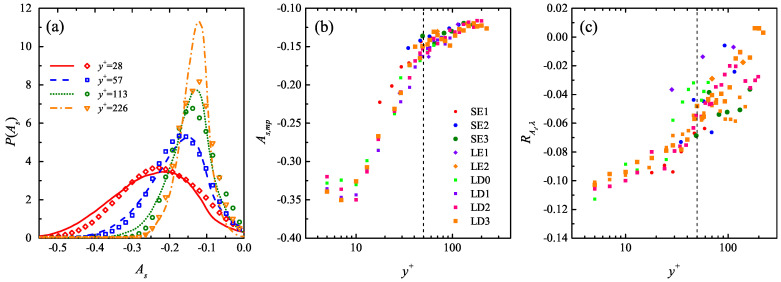
(**a**) Comparison of the PDF of the streak strength $A_{s}$ between the LE1 case (markers) and the LD1 cases (curves) at various $y^{+}$; (**b**) wall-normal variation of the most probable $A_{s,mp}$; (**c**) wall-normal variation of the correlation coefficient $R_{A_{s},\lambda }$ between $A_{s}$ and $\lambda^{+}$ in all the studied cases till y/δ=0.15.

Figure 11a shows the PDF of $A_{s}$ at typical $y^{+}$ in the LE1 and LD1 cases with equivalent $Re_{\tau }$. Both cases present similar $P(A_{s})$ profiles with a single peak and a long tail extending towards the left side. With the increase of $y^{+}$, a shrink of the spread of $A_{s}$ is seen, in together with the right shift of the most probable value $A_{s,mp}$. Figure 11b shows the wall-normal variation of $A_{s,mp}$ in all the studied cases. It reveals a minor growth of the magnitude of $A_{s,mp}$ below $y^{+}$ = 10, where $A_{s,mp}$ is mildly correlated with $Re_{\tau }$. This indicates both the active streak generation events in this region and the Re-dependent amplitude modulation effect that is consistent with the previous observation by Bradshaw and Langer [52]. Beyond $y^{+}$ = 10, $A_{s,mp}$ shows a constant decay till $y^{+}$ = 50, and then slowly asymptotes to the streak binarization threshold $u/\bar{U}=-0.1$ used in the streak identification algorithm.

The correlation coefficient $R_{A_{s},\lambda }$ between $A_{s}$ and $\lambda^{+}$, which measures the degree of the relationship between the strength of one streak and its spanwise spacing to the nearest neighborhood, are summarized in Figure 11c. A minor negative correlation, i.e., $R_{A_{s},\lambda }\approx -0.1$ is observed above the viscous sublayer, indicating that stronger streaks prefer to be spaced further away from its neighborhood. Such a correlation gradually relaxes towards $R_{A_{s},\lambda }$ = 0 in higher layers, and the relaxation rate sharply accelerates beyond $y^{+}$ = 50.

To our regards, the first fast growth stage of $\bar{\lambda^{+}}\left(y^{+}\right)$ below $y^{+}$ = 50 can be mainly attributed to the streak merging scenario. Smith and Metzler [30] proposed that the streak merging behavior is most active in the range of $10<y^{+}<30$. Tomkins and Adrian [41] observed that neighboring streaks merge with each other frequently at $20<y^{+}<100$, but the merging frequency remarkably drops beyond $y^{+}$ = 100. Note that for exponential decay of a variable, e.g., the streak population density $\Pi (y^{+})$ shown in Figure 10a, the decay rate is proportional to the variable’s magnitude. This is the case for the streak merging scenario: The more crowded streak distribution, the more chance for the occurrence of streak merging, thus leads to both the sharp reduction of Π and $A_{s}$ and the quick growth of $\lambda^{+}$.

Another attractive property of streak merging scenario is that it does not destroy the log-normal distribution of the streak spacing beyond the buffer region, which is clearly shown in Section 4.2. As stated by Smith and Metzler [30], ‘a random variable will develop a log-normal distribution when the independent influences cause variations which are proportional to the variable. Thus the log-normal distribution of streak spacing would seem to indicate that the independent physical influences which affect the variations in streak spacing are in some manner dependent up on the relative value of the streak spacing itself.’ On considering that the merging rate is strongly dependent on the streak spacing, the streak merging scenario, to our regards, might be a possible candidate for such ‘physical influences’.

For a quantitative description, the streak merging events are counted from instantaneous snapshots as where a pattern of two neighboring low-speed streak skeletons converging into one is identified. The related detection algorithm is briefly described in Appendix B.1. Figure 10b summarizes the merging frequency $\rho_{m}/\Pi$, in which $\rho_{m}$ is the average number of the streak merging event per unit span. Figure 10c further shows the ratio between the streak merging and splitting frequency $\rho_{m}/\rho_{s}$, the latter is counted via a similar scheme. It is clearly shown that $\rho_{m}/\Pi \left(y^{+}\right)$ of all the studied cases follow a two-sectional decay gapped at about $y^{+}$ = 50, similar to that of $\Pi (y^{+})$. This is consistent with the observation of Smith and Metzler [30] and Tomkins and Adrian [41], and highlights a strong correlation between the streak population and the streak merging frequency: the amalgamation of two neighboring streaks will leave the signature of only one streak in higher layer; as a consequence, the increased streak spacing there will lower the local streak merging frequency.

Interestingly, the streak splitting event, which serves as a counter-acting role of inhibiting the streak spacing growth, has a slightly higher frequency than that of the streak merging event in the near-wall region. However, such an in-equilibrium gradually diminishes with the increase of $y^{+}$. A detailed examination of instantaneous velocity fields show that new-born streaks through streak splitting always have comparably weaker strength and shorter length; while in a streak merging event, the merged streak tends to pose weaker peak strength but broader width. Therefore, both events contribute to the wall-normal decay of the streak strength, and the latter weighs more to promote the quick growth of the streak spacing in the near-wall region.

For those streaks with stronger strength and gaped further away from others, they have more chance to survive through the active streak instability process in the near-wall region. Recalling that the second stage of the empirical streak spacing model presents a linear growth of μ with reduced slope but a quasi-constant $\sigma^{2}$ beyond $y^{+}$ = 100. Since $\sigma^{2}$ characterizes the width of the $P(\lambda^{+})$ profile, the constant $\sigma^{2}$ implies a passive streak dynamics in this region: due to the rather large streak spacing, the streak merging/splitting in higher flow layer is inactive; instead, those small-scale streak-liked structures, most of which might be the remnants of near-wall streaks, act as being ‘frozen’, i.e., they can be either synchronized to larger scales to form the core region of LSMs or gradually dissipated by viscosity.

## 5. Synthetic Simulation of the Spanwise Spectra

In this section, we attempt to restore part of the spanwise spectra of *u* through synthetic simulation by only considering the spanwise distribution of streaks that is independent of Re. One of the practical meanings of this attempt is that it might promote the understandings on how large-scale structures affects the spectra to formulate a Re-dependency, and it might provide useful information for the development of the near-wall model in LES.

The idea is to randomly distribute multiple elementary streak units along spanwise direction with spacing determined by the empirical model developed in Section 4.2. For simplicity, only 1D scenario, i.e., the spanwise variation of the *u* component fluctuation velocity, is considered here. The elementary streak unit follows the model proposed by Hutchins and Marusic [43]:

(9) $$\theta \left(z_{i}\right)=\pi z_{i}/\lambda_{z},-\frac{3}{2}\lambda_{z}<z_{i}<\frac{3}{2}\lambda_{z},$$

(10) $$u_{s}\left(\theta \left(z_{i}\right)\right)=A_{s}\left(-\frac{3}{4}-\frac{1}{4}\mathrm{sgn}\left(\cos\left(\theta \right)\right)\right)\cos\left(\theta \right).$$

**Figure 12:**
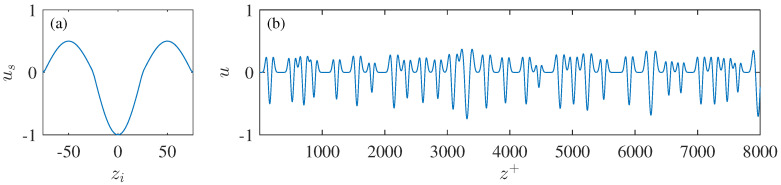
(**a**) Profile of the elementary streak unit described by Equations (9) and (10); (**b**) an example of a synthetic signal of $u(z^{+})$ with ${\bar{\lambda }}_{z}^{+}$ = 100 and $A_{s}$ = 1.

In this model, $\theta (z_{i})$ is the phase angle at $z_{i}$; $u_{s}\left(z_{i}\right)$, which is actually a 3/2 periods of cosinusoid modulated by a box function, represents a spanwise profile of one single low-speed streak centering at $z_{i}$ = 0; $\lambda_{z}$ sets the wavelength of the streak; $A_{s}$ is the nominal streak amplitude and sgn(cos(θ)) returns the sign of cos(θ). Figure 12a shows a typical streak unit with $\lambda_{z}^{+}=100$ and $A_{s}$ = 1. For a given $y^{+}$, multiple streak units whose $\lambda_{z}^{+}$ are randomly generated via the empirical streak spacing model of Equations (3), (7), and (8) are successively added along the spanwise direction till the whole span is full, i.e.,

(11) $$u\left(z^{+}\right)=\sum_{i=1}^{N}u_{s,i}\left(z^{+}-z_{i}^{+}\right),z^{+}\in \left[1,2^{13}\right].$$

in which $u_{s,i}$ is the *i*th streak unit centering at $z_{i}$ with wavelength of $\lambda_{zi}$, and $u(z^{+})$ is the full signal with a total length of $2^{13}$ wall units. To avoid severe overlap which causes unexpected wavelength growth, one streak is gaped from its neighborhoods by the following constraint:

(12) $${\left(z_{i}^{+}-z_{i-1}^{+}\right)}^{2}\geq \lambda_{z,i}^{+2}\;\mathrm{and}\;{\left(z_{i}^{+}-z_{i+1}^{+}\right)}^{2}\geq \lambda_{z,i}^{+2}.$$

Finally, a Gaussian smooth is applied to $u(z^{+})$ to eliminate discontinuity. An example of the $u(z^{+})$ profile is shown in Figure 12b with ${\bar{\lambda }}_{z}^{+}=100$ and $A_{s}$ = 1. Section 4.2 already shows that the streak amplitude $A_{s}$ is only weakly correlated with the streak spacing λ in the near-wall region. Here, we assume $A_{s}$ to be constant at each flow layer with magnitude equal to the local $u_{rms}$. This actually attributes all the *u* component TKE to small-scale streaks and ignores the TKE contribution from either large-scale structures or their modulation effect on smaller ones. Although this assumption is far from the real case, it provides an artificial scenario to infer the effect of the unconsidered large-scale motions on the velocity spectra.

**Figure 13:**
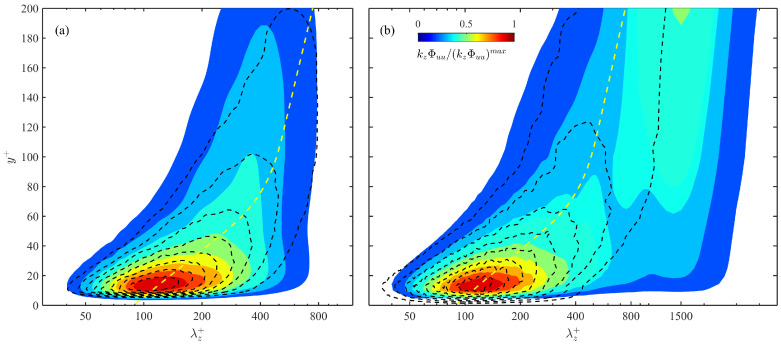
Comparison of the premultiplied spanwise spectra $k_{z}\Phi_{uu}$ simulated by the simplified synthetic method (dashed isolines) with the original one (pseudo-color maps) in (**a**) the LD0 case and (**b**) the LD3 case. $k_{z}\Phi_{uu}\left(\lambda_{z}^{+}\right)$ are all normalized by the maximum value in the near-wall region, and the contour labels are evenly spaced from 0.2 to 0.9 with interval of 0.1. Dashed lines are ${\bar{\lambda }}^{+}$ predicted by the empirical model in Section 4.2.

Figure 13 compares the $k_{z}\Phi_{uu}$ spectra of the fabricated $u(z^{+})$ fields (dashed isolines) to the original ones (pseudo-color maps) in the LD0 and LD3 cases. Combing with other cases that are not shown, it can be concluded that the present simulation, despite its simplicity, is capable of restoring the core region of the inner-layer spectra patch within $y^{+}<50$ and $\lambda_{z}^{+}<300$. The reason, to our regards, is that the ridge of the inner-layer spectra patch is well predicted by the empirical streak spacing model, which in turn is fully utilized when modeling the $u(z^{+})$ fields. More interestingly, Figure 13a show that if the outer-layer spectra patch is absent, the general shape of $k_{z}\Phi_{uu}$ can be acceptably captured till $y^{+}\approx 100$. This describes a scenario of the penetration of small-scale streaks into higher layer, which is further supported by the observation that with the presence of the outer-layer spectra patch, the small-scale part of the $k_{z}\Phi_{uu}$ spectra on the left side of the mean streak spacing (bold dashed lines in Figure 13b) is roughly predicted till $y^{+}\approx 100$.

Since LSMs and their near-wall footprints are not considered in the present synthetic simulation, the yielded $k_{z}\Phi_{uu}$ significantly differs from the original spectra in the large-scale domain with $\lambda_{z}^{+}>400$, as is shown in Figure 13. One can get an impression on the Re-dependency of these large-scale structures by subtracting the simulated $k_{z}\Phi_{uu}\left(\lambda_{z}^{+}\right)$ profiles from the original ones. Recalling that the full *u* component TKE (measured as $u_{rms}$), part of which is originally carried by large-scale structures, is arbitrarily assigned to small-scale streaks during the fabrication of $u(z^{+})$, this leads to an overestimation of the energy content in the small-scale near-wall domain, which becomes more prominent at higher Re (comparing the near-wall profiles in Figure 13a,b for an illustration).

Such an overestimation might be improved by assigning not the whole $u_{rms}$ but the streak-contributed portion of $u_{rms}$ to $A_{s}$. A scale-based decomposition, instead of the energy-based POD filtering used in Section 3, is thus needed to quantify the TKE contribution from streaks. This is an issue to be studied in the future. Nevertheless, on considering that the present synthetic simulation only relies on the knowledge of both the $u_{rms}\left(y\right)$ profile that is dependent on Re and the streak spacing distribution that seems to be independent of Re, the slight difference in the simulated small-scale energy content is acceptable, and will not undermine the practical value of such a test. Of course, more complicated issues, like accounting streaks’ streamwise extent and modeling both the dynamical process of the streak instability [4] and their response to outer-layer large-scale structures [54,55], should be taken into consideration. But one of the particular attractions of the present idea is that due to the Re-independence of the streak distribution, the modeling of the streak dynamics might be obtained from a low-Re DNS database via either the techinque of reduced-order modeling [100,101] or minimum flow unit simulation [102], and then applied to high-Re case through proper scaling.

## 6. Concluding Remarks

In summary, the present work deals with the wall-normal variation of the characteristic lateral length scale of small-scale streak-liked structures in a smooth-wall turbulent boundary layer. The primary aim is to extend the existing knowledge on the streak spacing in the near-wall region to higher flow layers. Morphological analysis on the *u* component fluctuating velocity is taken in a range of $Re_{\tau }$ = 440∼2400. It is found that the streak spacing λ keeps a log-normal distribution till the upper bound of the log layer. The inner-scaled mean and most probable value, i.e., ${\bar{\lambda }}^{+}$ and $\lambda_{mp}^{+}$, follows a two-stage exponential wall-normal growth that is less dependent on Re and can be well described by a two-sectional empirical model.

The first fast growth stage of ${\bar{\lambda }}^{+}\left(y^{+}\right)$ and $\lambda_{mp}^{+}\left(y^{+}\right)$ below $y^{+}$ = 50 can be attributed to the active streak merging event, which results in a quick drop of both the streak strength and the streak population density there. A simplified synthetic simulation, which only models the spanwise distribution of streak elements via the proposed empirical model, fairly restores the core region of the inner-layer $k_{z}\Phi_{uu}$ spectra patch residing in this region. The second stage beyond $y^{+}$ = 50 presents a reduced growth rate in ${\bar{\lambda }}^{+}\left(y^{+}\right)$ and $\lambda_{mp}^{+}\left(y^{+}\right)$, consistent with the relaxation of the decay of the streak strength, the streak population density and the streak merging frequency. This suggests that most of the small-scale streaks identified beyond the buffer layer might be the remnants of near-wall structures. Despite of their sparse population, they contribute to the small-scale part of the $k_{z}\Phi_{uu}$ spectra till $y^{+}$ = 100, which can be fairly restored by the simplified synthetic simulation.

To our regards, the exponential scaling of the streak spacing proposed here, i.e., $y^{+}\propto \ln{\bar{\lambda }}^{+}$ and $y^{+}\propto \ln\lambda_{mp}^{+}$ till the upper bound of the log layer, is different from the linear scaling of wall-attached large-scale structures [11,41,67,68]. This suggests that small-scale streaks do not behave in an attached-eddy way. Instead, those structures that survive through the active near-wall streak instability events passively lift to higher layers, either gradually fading out due to viscous dissipation or being synchronized into larger-scale structures. It is believed that more detailed information in this aspect will provide helpful insight into the origin of LSMs, and thus deserves to be studied later.

Finally, since the Re-independency of the streak spacing suggests that the amplitude modulation does not alter the geometric characteristics of small-scale structures, this provides a justification for the so-called ‘universal’ signal that was used by Marusic et al. [55] and Zhang and Chernyshenko [103] to predict the near-wall fluctuating velocity statistics given the knowledge of the log-layer large-scale signal. Nevertheless, the failure of restoring the large-scale part of $k_{z}\Phi_{uu}$ in the simplified synthetic simulation stresses the accumulated importance of large-scale motions with the increase of Re. To fully restore the whole spectra, the geometrical characteristics of these large-scale motions should be modeled properly. Note that the scale separation tool and the morphological identification algorithm used in the present study can be also applied for such a purpose in the future.

## Figures and Tables

**Table 1:** Summary of previous studies on the spanwise spacing of low-speed streaks.

Studies	Flow Type	Reτ	$y^{+}$	${\bar{\lambda }}^{+}$	$\Delta Z^{+}$	Distribution	Method
Coantic [32]	Pipe flow	2500 ($Re_{\theta }$)	$y^{+}<5$	110–130	−	−	Hot-wire with correlation analysis
Schraub and Kline [72]	Boundary layer	501	$y^{+}<5$	100±20	−	−	Dye and $H_{2}$ bubble visualization
Kline et al. [29]	Boundary layer	431, 501	$y^{+}\approx 2$	91, 106	500	−	Dye and $H_{2}$ bubble visualization
Bakewell Jr and Lumley [73]	Boundary layer	∼239	$y^{+}\;=$ 0–7	80–100	−	−	Hot-wire with space-time correlation
Gupta et al. [33]	Boundary layer	870–2160	$y^{+}=$ 3.4–10.8	97.5–151.2	373	−	Hot-wire with short duration correlation
Lee et al. [34]	Pipe flow	1735–2045 ($Re_{\theta }$)	$y^{+}<0.5$	105-107	250	Lognormal	Electrochemical measurement with spatial correlation
Nakagawa and Nezu [36]	Channel flow	318, 696	$y^{+}=$ 10–100	100–1000	3000	Lognormal	Hot-wire with conditional correlation
Smith and Metzler [30]	Boundary layer	1040	$y^{+}=$ 1–30	93–146	1000	Lognormal	Hydrogen bubbles visualization
Kim et al. [74]	Channel flow	180	$y^{+}=$ 1–23	100–125	1150	−	Averaged correlation
Klewicki et al. [35]	Atmospheric surface layer	$3\times 10^{5}$	$y^{+}=3.4$	93.1	−	−	Fog visualization
Lagraa et al. [75]	Boundary layer	1170	$y^{+}=$ 0–50	100–180	216	−	Electrochemical measurement with space-time correlation
Lin et al. [76]	Boundary layer	7800 ($Re_{\theta }$)	$y^{+}=$ 15–50	110–120	320	Rayleigh	Stereo-PIV with morphological analysis

**Table 2:** Summarization of characteristic boundary layer parameters. SE1∼SE3 are small-field-of-view (FOV) particle image velocimetry (PIV) cases; LE1 and LE2 are large-FOV PIV cases; LD0∼LD3 are large-FOV direct numerical simulation (DNS) cases from Simens et al. [89] and Sillero et al. [90,91].

Cases	$U_{\infty }$	Reθ	δ	*H*	$u_{\tau }$	Reτ	FOV	Spatial Res.	${\mathrm{Tu}}_{\tau }/\delta$	Marker
	(mm/s)		(mm)		(mm/s)		$\Delta X^{+}\times \Delta Z^{+}$	$\Delta x^{+}\times \Delta z^{+}$		
SE1	146	908	75.5	1.46	6.7	444	480×600	6 × 6	43	•
SE2	299	2044	65.8	1.39	13.1	761	400×500	5 × 5	97	•
SE3	455	3125	62.1	1.37	18.6	1014	560×700	7 × 7	144	•
LE1	145	2983	202	1.32	5.6	1135	4000×1500	9 × 9	22	◆
LE2	340	5076	174	1.30	13.7	2431	8900×3750	23 × 23	57	◆
							(8900×1500)			
LD0	999	945	2.6	1.43	47.8	440	2000×1500	6 × 4	-	■
LD1	1001	3100	7.6	1.38	40.3	1100	2000×1500	7 × 4	-	■
LD2	1002	4800	11.4	1.37	38.1	1500	2000×1500	7 × 4	-	■
LD3	1001	6500	15.4	1.36	36.8	2000	2000×1500	7 × 4	-	■

**Table 3:** Summarization of wall-parallel planes being studied. Those planes at y/δ>0.15 are indicated by asterisks.

Case	Reτ	$\Delta Z^{+}$	Wall-Normal Height $y^{+}$											
SE1	444	600	17	24	29	35	47	59	76 *	94 *	118 *	147 *	182 *	223 *
SE2	751	500				35	46	58	70	93	116 *	150 *	185 *	231 *
SE3	1014	700					49	65	81	98	131	163 *		212 *
LE1	1135	1500				28		57			113			226 *
LE2	2431	1500							70			140		280
LD0∼LD3	440∼2000	1500	5∼223											

**Figure A1:**
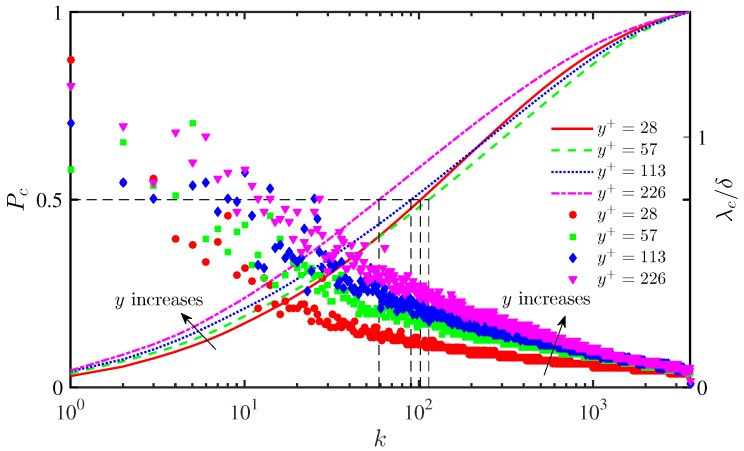
The cumulative turbulent kinetic energy (TKE) contribution $P_{n}$ (lines) and the characteristic spanwise scale $\lambda_{c}^{\phi_{n}}$ (solid symbols) carried by each POD mode at various $y^{+}$ in the LE1 case.

**Figure A2:**
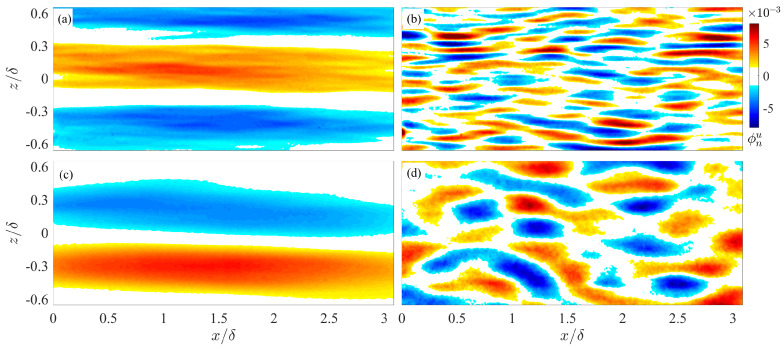
Typical mode basis function $\phi_{n}^{u}$ in the LE1 case. (**a**) *n* = 1, $y^{+}$ = 28; (**b**) *n* = *s* = 100, $y^{+}$ = 28; (**c**) *n* = 1, $y^{+}$ = 226; (**d**) *n* = *s* = 61, $y^{+}$ = 226. In (**b**,**d**), the mode rank *s* is chosen to make the POD energy cutoff threshold $P_{s}$ = 0.5.

**Figure A3:**
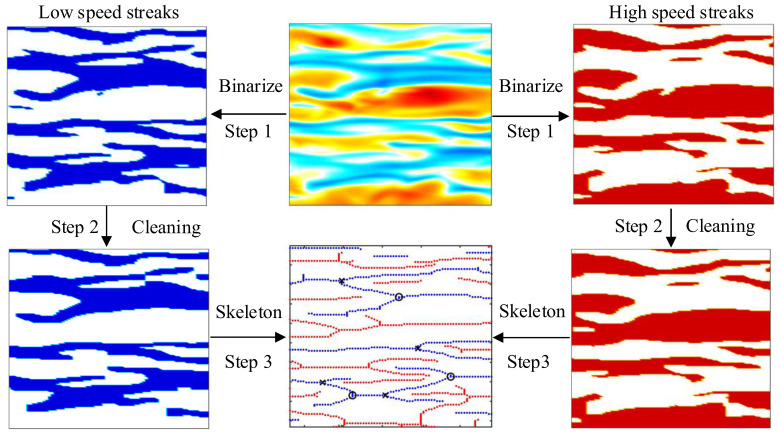
Schematic illustration of the morphological-based streak identification algorithm. Circle and cross makers in the final streak skeleton subplot indicate the detected streak merging and splitting event, respectively.

**Figure A4:**
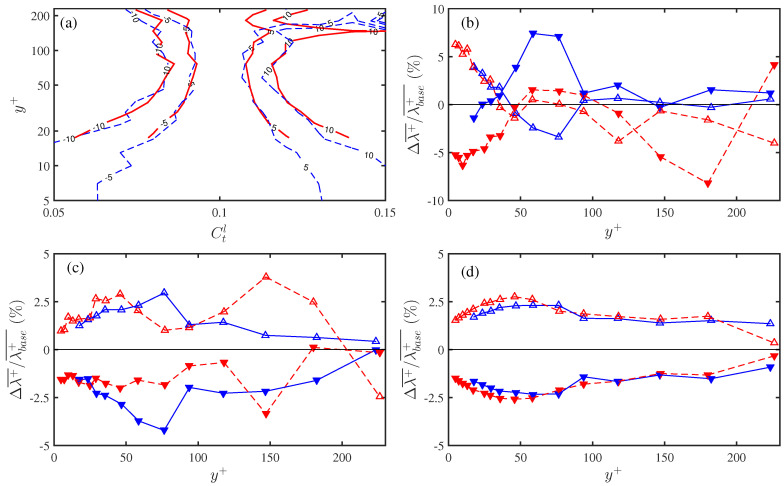
Percentage of the change of the mean streak spacing ${\bar{\lambda }}^{+}$ from the baseline case due to the change of the following parameters in the morphological algorithm: (**a**) the binary threshold $C_{t}^{l}$; (**b**) the size of the cleaning structure element, baseline 48×8, △72×12 and ▾ 24×4 wall units${}^{2}$; (**c**) the area cutting-off threshold, baseline 800, △ 400, ▾ 1200 wall units${}^{2}$ and (**d**) the spur length threshold, baseline 12, △ 20, ▾ 4 wall units. In (**a**), solid and dashed isolines indicate the SE1 case and the LD1 case, respectively; in (**b**–**d**), markers with solid lines indicate the SE1 case and markers with dashed lines the LD1 case.

**Figure A5:**
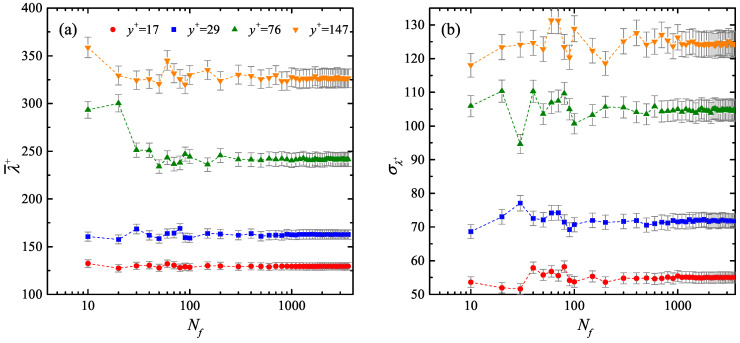
Effect of the analyzed frame number $N_{f}$ on (**a**) the mean streak spacing ${\bar{\lambda }}^{+}$ and (**b**) the r.m.s of the streak spacing $\sigma_{\lambda^{+}}$ in the SE1 case at • $y^{+}$ = 17; ■ $y^{+}$ = 29; ▲ $y^{+}$ = 76; ▾ $y^{+}$ = 147. Error bars indicate ±3% variation.

**Figure A6:**
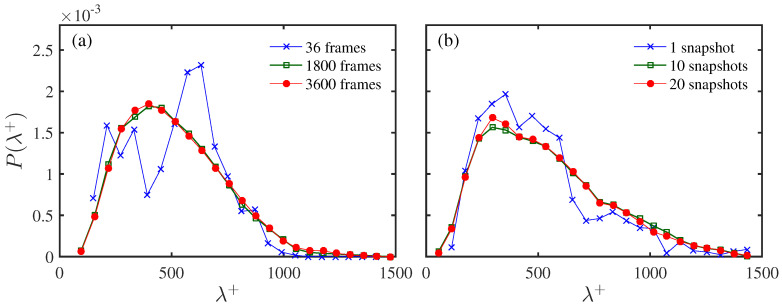
Effect of the analyzed frame/snapshot number on the shape of $P(\lambda^{+})$ in (**a**) the LE2 case at $y^{+}=140$ and (**b**) the LD3 case at $y^{+}=147$. In (**a**), ×36 frames, □ 1800 frames, • 3600 frames; in (**b**), ×1 snapshot, □ 10 snapshots, • 20 snapshots. Note that for the LE2 case, 1800 instantaneous frames lead to more than $10^{5}$ samples of streak spacing. While in the LD3 case, 1 snapshot of the DNS velocity field is sliced into 10 frames with FOV of 2000 × 1500 wall units, the total number of 20 DNS snapshots correspond to 200 frames, with more than $10^{4}$ samples being recorded.

## Appendix A. Scale and Mode Shape of POD Modes

Snapshot POD is used to decompose the fluctuating velocity fields into discrete POD modes. It has been checked that the ensemble size of the SE and LE cases are large enough for a converged decomposition. Since POD is based on an optimal energy recovery criteria, the relationship between the energy content of POD modes and their characteristic length scale should be checked before POD method can be used for scale separation.

The characteristic spanwise scale $\lambda_{c}^{\phi_{n}}$ carried by the *n*th rank POD mode can be estimated from $R_{\phi_{n}^{u}\phi_{n}^{u}}\left(r_{z}\right)$, the two-point correlation of the *u* component mode basis function $\phi_{n}^{u}$. In the present work, $\lambda_{c}^{\phi_{n}}$ is defined as the gap between two negative valleys in the $R_{\phi_{n}^{u}\phi_{n}^{u}}\left(r_{z}\right)$ map, similar to $\bar{\lambda_{z}^{+}}$ illustrated in Figure 4. Figure A1 shows $\lambda_{c}^{\phi_{n}}$ as a function of the mode rank *n* at typical $y^{+}$ in the LE1 case, in together with the cumulative TKE contribution curve $P_{n}\left(n\right)$. In general, $\lambda_{c}^{\phi_{n}}$ monotonically decreases with the increase of *n*, indicating a distinct correlation between the energy content and the length scale in the hierarchy of POD mode set.

$\phi_{n}^{u}$ of the first POD mode (*n* = 1) at $y^{+}$ = 28 and 226 in the LE1 case are illustrated in Figure A2a,c. Both characterize high- and low-speed strips aligned in spanwise direction with quasi-periodicity. Their spanwise scales are O(δ), and the streamwise coherence extends beyond 3δ. On considering its TKE significance, the first POD mode is regarded as the projection of LSMs onto the mode subspace. The geometrical similarity of $\phi_{n=1}^{u}$ between the inner layer ($y^{+}$ = 28) and the outer layer ($y^{+}$ = 226) implies a scale invariance when LSMs extend their influence into the near-wall region, consistent with the outer-layer spectra patch shown in Figure 13b.

As a comparison, Figure A2b,d show $\phi_{s}^{u}$ of a *s*th rank POD mode whose $P_{s}$ is 0.5 at $y^{+}$ = 28 and 226, respectively. Recalling that $P_{s}$ = 0.5 is the POD energy cutoff threshold used in Section 3. Figure A1 shows that the mode rank *s* with $P_{s}$ = 0.5 decreases with the increase of $y^{+}$, the corresponding $\lambda_{c}^{\phi_{s}}$ increases with $y^{+}$, but the magnitude is always far from O(δ). Such a small-scale feature can be evidenced by Figure A2b,d, where small-scale streaky pattern of $\phi_{s}^{u}$ is observed in both inner and outer layer, in together with a clear tendency of the scale growth. Finally, it can be concluded from Figure A1 that the leading-order $V^{L}$ fields constructed in Section 3 includes all the POD modes whose spanwise scales are larger than δ.

Other SE and LE cases reveal a similar relation between the TKE content and the length scale in the POD mode set. The only difference is that the streaky pattern in the leading-order POD modes in the SE cases poses smaller length scale due to the FOV limitation.

## Appendix B. Morphological-Based Streak Identification Algorithm

### Appendix B.1. Algorithm Description

The morphological method used in Section 4 identifies streak-liked structures from instantaneous *u* component field. As shown in Figure A3, this algorithm poses 3 steps: binarization, cleaning, and skeletonization.

Step 1, binarization. According to the definition of streak described in Section 1, the instantaneous *u* component fluctuating velocity in wall-parallel *x*-*z* plane is binarized into low- and high-speed elements $F_{i}^{l}$ and $F_{i}^{h}$ by:

(A1) $$F_{i}^{l}=\left\{\begin{array}{cc} 1, & F_{d}<-C_{t}^{l}\\ 0, & \mathrm{Otherwise} \end{array},\right.F_{i}^{h}=\left\{\begin{array}{cc} 1, & F_{d}>C_{t}^{h}\\ 0, & \mathrm{Otherwise} \end{array}.\right.$$

The detection function $F_{d}$ in Equation (A1) is the ratio of the fluctuating velocity to the mean velocity of the investigated flow layer: $F_{d}=u/\bar{U}$. Following previous studies [41,46,48,104], the streak strength threshold $C_{t}^{l}$ in Equation (A1) was set to be 0.1. And the ratio between $C_{t}^{h}$ and $C_{t}^{l}$ was fixed as 0.5 since Smith and Metzler [30] reported that the momentum flux ratio between high- and low-speed streaks was about 0.5.

Step 2, cleaning. A combination of closing and opening operation, based on a rectangle filter template, is applied onto $F^{l}$ and $F^{h}$ to fill small holes and remove isolated noise. To simulate the large aspect ratio of streaks, the length/width of this filter template is set to be 50/10 wall units, the same as in Lin et al. [76]. Those connecting regions in $F^{l}$ whose aspect ratio is smaller than 2 are also discarded, this guarantees that only streamwise elongated connecting regions are considered. After the closing and opening operation, there are still some connecting regions in $F^{l}$ too small to be taken as streaks. An area cutting-off threshold of 800 wall units${}^{2}$ is then set to reject these small structures.

Step 3, skeletonization. A simple morphological skeleton operation widely used in computer vision [105] is taken to shrink the identified structures into a skeleton. This operation will generate unexpected branches whose length scale is quite small compared to that of the main stem. The spur operation is then followed to trim the branches whose length is smaller than 12 wall units.

The above three procedures are conceptually similar to that of Lin et al. [76]. An additional concern here is that some streaks might not be well separated from others due to the streak merging or splitting event; moreover, the branched pattern can be also caused by the skeleton operation, as mentioned above. Therefore, the location of high-speed streaks (or high-speed regions in higher layers) are used as a supplemental criterion: the gap between two neighboring low-speed skeletons is counted only if there is at least one high-speed skeleton between them. This is consistent with Smith and Metzler [30], who considered streaks to be completely merged if there is no high-speed region between them.

Finally, on considering the possibility that the streaks close to the FOV edge are incompletely captured, the skeletons crossing the streamwise boundary of the FOV are cut-off by a length of 50 wall units. In a similar sense, the skeletons whose distance to the spanwise FOV extent are smaller than 20 wall units are also discarded. The influence of the parameters on the statistics of the streak spacing will be further discussed in Appendix B.2.

After the streak skeleton identification, the streak merging and splitting event can be detected by examining the topology around a node on one streak skeleton where a new branch grows towards either downstream or upstream. Those branches shorter than 50 wall units, which might be caused by local expansion of streaks, are rejected as an additional streak skeleton. The streak skeleton nodes are detected by a connectivity evaluation scheme that is commonly used in computer vision [105]. An example of the detected streak merging/splitting events is shown in Figure A3 for qualitative evidence of the feasibility of this detecting method.

### Appendix B.2. Effect of Algorithm Parameters on the Streak Spacing Statistics

In the present morphological algorithm, several parameters need to be manually selected; therefore, their influence on the streak identification should be evaluated carefully. The default baseline parameters are set as: the binary threshold $C_{t}^{l}=0.1$, the size of cleaning structure element 50×10 wall units, the spur length 12 wall units and the area cutting-off threshold 800 wall units${}^{2}$. This parameter set is used in Section 4 for the streak identification. The effect of each parameter is then inspected by examining the relative change of the mean streak spacing ${\bar{\lambda }}^{+}$ from the baseline case. Here, only the SE1 and LD1 cases are illustrated, all the other cases pose similar behavior and are not presented. In the following test, the spanwise FOV span of the LD1 case is truncated to $\Delta Z^{+}$ = 600 for a direct comparison with the SE1 case.

The streak strength threshold $C_{t}^{l}$ defines the boundary of the low-momentum region, and thus directly determines the size and population of the identified streaky structures. Figure A4a gives the relative deviation of ${\bar{\lambda }}^{+}$ from the baseline case ($C_{t}^{l}=0.1$) as $C_{t}^{l}$ varying from 0.05 to 0.15. For flow layers beneath $y^{+}=10$, ${\bar{\lambda }}^{+}$ is only weakly correlated with $C_{t}^{l}$; beyond that, the dependency becomes a bit more strong: ±25% variation of $C_{t}^{l}$ results in about ±10% variation of ${\bar{\lambda }}^{+}$. Moreover, the correlation between ${\bar{\lambda }}^{+}$ and $C_{t}^{l}$ quantitatively holds for all the higher layers, this makes the trend of the wall-normal growth of ${\bar{\lambda }}^{+}$ decoupled from the selection of $C_{t}^{l}$.

Except for $C_{t}^{l}$, all the other parameters pose little influence on ${\bar{\lambda }}^{+}$. As shown in Figure A4b, the cleaning structure elements with two different sizes, 24×4 and 72×12 wall units, are tested. They only result in ±6% variation of ${\bar{\lambda }}^{+}$ from the baseline case. Two different area cutting-off thresholds of 400 and 1200 wall uints${}^{2}$ (with ±50% variation) make the relative change of ${\bar{\lambda }}^{+}$ within ±3%, as shown in Figure A4c. Finally, Figure A4d shows that in the procedure of removing small branches from the main stem of the streak skeleton, ±66% variation of the spur length threshold leads to a variation of ${\bar{\lambda }}^{+}$ smaller than ±2%.

In short, only the choice of $C_{t}^{l}$ has a relatively large influence on the streak spacing statistics. The default value of $C_{t}^{l}=0.1$ is chosen to be consistent with a majority of previous researches [41,46,48,104].

### Appendix B.3. Effect of Ensemble Size on the Streak Spacing Statistics

The convergence state of the streak spacing is essential for estimating the related streak statistics. The dependency of the first and second-order statistics of the streak spacing, i.e., ${\bar{\lambda }}^{+}$ and $\sigma_{\lambda^{+}}$, on the number of the analyzed frames $N_{f}$ is shown in Figure A5 at four $y^{+}$ in the SE1 case. Note that ${\bar{\lambda }}^{+}$ and $\sigma_{\lambda^{+}}$ is directly calculated from the whole ensemble, instead of being estimated by the log-normal fitting in Section 4.1. It is clearly shown that more snapshots are needed for a stable ${\bar{\lambda }}^{+}$ and $\sigma_{\lambda^{+}}$ at higher flow layer. This is reasonable since the streak population decays with the increase of $y^{+}$, as is shown in Figure 10. The convergence of $\sigma_{\lambda^{+}}$ is relatively slower than that of ${\bar{\lambda }}^{+}$, but a total ensemble size of around 3000 frames is sufficient. Moreover, it reminds us that careful inspection of the convergence of the PDF profile $P(\lambda^{+})$ is critical. Figure A6 compares the insufficiently sampled $P(\lambda^{+})$ profiles with the converged ones in both the LE2 and LD3 cases. It is evidenced that half the total ensemble is enough to yield a converged $P(\lambda^{+})$ in the log layer ($y^{+}\approx$ 140). The low-sampled profiles present multi-modal shape; however, increasing the ensemble size will smooth all the non-physical PDF peaks and form a single-peak left-skewed shape. Using this inspection, we have checked that $P(\lambda^{+})$ gets acceptable convergence till $y^{+}\approx 220$ in all the studied cases.
